# Testudines as Sentinels for Monitoring the Dissemination of Antibiotic Resistance in Marine Environments: An Integrative Review

**DOI:** 10.3390/antibiotics10070775

**Published:** 2021-06-25

**Authors:** Kezia Drane, Roger Huerlimann, Michelle Power, Anna Whelan, Ellen Ariel, Madoc Sheehan, Robert Kinobe

**Affiliations:** 1 Centre for Molecular Therapeutics, College of Public Health, Medical and Veterinary Sciences, Australian Institute of Tropical Health and Medicine, James Cook University, Townsville, QLD 4811, Australia; Ellen.ariel@jcu.edu.au; 2 Centre for Sustainable Tropical Fisheries and Aquaculture, Centre for Tropical Bioinformatics and Molecular Biology, College of Science and Engineering, James Cook University, Townsville, QLD 4811, Australia; Roger.huerlimann@jcu.edu.au; 3 Department of Biological Sciences, Macquarie University, Sydney, NSW 2109, Australia; michelle.power@mq.edu.au; 4 Townsville Water and Waste, Wastewater Operations, Townsville, QLD 4810, Australia; aw3@townsville.qld.gov.au; 5 College of Science, Technology and Engineering, James Cook University, Townsville, QLD 4811, Australia; Madoc.sheehan@jcu.edu.au

**Keywords:** anthropogenic pollution, antimicrobial pollution, multidrug-resistant bacteria, horizontal gene transfer, wastewater treatment plants, turtle rehabilitation centres

## Abstract

Dissemination of antibiotic resistance (AR) in marine environments is a global concern with a propensity to affect public health and many ecosystems worldwide. We evaluated the use of sea turtles as sentinel species for monitoring AR in marine environments. In this field, antibiotic-resistant bacteria have been commonly identified by using standard culture and sensitivity tests, leading to an overrepresentation of specific, culturable bacterial classes in the available literature. AR was detected against all major antibiotic classes, but the highest cumulative global frequency of resistance in all represented geographical sites was against the beta-lactam class by a two-fold difference compared to all other antibiotics. Wastewater facilities and turtle rehabilitation centres were associated with higher incidences of multidrug-resistant bacteria (MDRB) accounting for an average of 58% and 49% of resistant isolates, respectively. Furthermore, a relatively similar prevalence of MDRB was seen in all studied locations. These data suggest that anthropogenically driven selection pressures for the development of AR in sea turtles and marine environments are relatively similar worldwide. There is a need, however, to establish direct demonstrable associations between AR in sea turtles in their respective marine environments with wastewater facilities and other anthropogenic activities worldwide.

## 1. Introduction

Antibiotics have provided effective and safe treatments for bacterial infections and are an essential tool in modern medicine [1]. Unfortunately, the rise in antibiotic resistance (AR) among common bacterial pathogens is of increasing concern, and infections caused by multiple drug-resistant bacteria (MDRB) are associated with an increased frequency of mortality [2,3]. To address this problem, the World Health Organisation has commissioned a series of action plans including increasing the awareness of the magnitude and impact of AR through communication, training, education, surveillance, and research [4]. Dissemination and propagation of AR in marine environments is also an increasing concern, as this may affect previously unpolluted microbial communities within the world’s oceans [5,6]. In effect, a higher prevalence of MDRB in marine environments threatens marine wildlife but also serves as a platform for potential transmission of environmental antibiotic-resistant genes (ARGs) to human pathogens. For instance, AR and virulence genes that are characteristically linked with methicillin-resistant *Staphylococcus aureus* were isolated in healthy edible marine fish in South Africa [7]. Similarly, another review [8] reported examples of infection and disease caused by *Vibrio* spp. and *Salmonella* spp. in humans following the consumption of sea turtle meat and eggs. Despite the existence of these specific examples pertaining to the threat of AR in the marine environment, our comprehensive understanding of ecological and anthropologic factors that contribute to the development and dissemination of AR in such environments is lacking. To study and elucidate factors that contribute to AR in otherwise very diverse and complex marine ecosystems, sentinel animal species whose health is considered to reflect the wellbeing of these ecosystems should be used. Sea turtles are considered a good sentinel species for monitoring the health of marine ecosystems; they have been used in many toxicological and trophic ecology studies [9,10]. This value as sentinels is due to their long lifespan and tendency to bioaccumulate pathogens and contaminants including antibiotics [11,12,13]. Furthermore, antibiotic-resistant bacteria (ARB) have been previously described as a bio-indicator of anthropogenic pollution within marine environments [11,13,14]. However, no study has provided a critical integrative evaluation of the role played by sea turtles in monitoring the prevalence, or dissemination of AR in marine environments globally. In this review, we provide a critical appraisal on the utilization of turtles as sentinels in determining causes, prevalence, and distribution patterns of AR in marine ecosystems.

### 1.1. General Mechanisms by Which Bacteria Develops Antibiotic Resistance

Many of the antibiotic compounds used in medicine and agriculture are naturally occurring, and therefore, co-existing bacteria have evolved mechanisms to survive exposure to antibiotics or other environmental threats [13]. This means that bacterial populations are often intrinsically resistant to one or more antibiotics [15]. General mechanisms by which bacteria become intrinsically resistant to antibiotics include the alteration of the target sites, adjustment or destruction of the antibiotic molecule, and removal of the antibiotic molecule via efflux transporters, or reduced influx of the antibiotic through decreased membrane permeability [15]. Mutation in genes that code for these molecular targets of antibiotic compounds allows the mutated bacterial cell to survive exposure to the antibiotic compound where other cells would be susceptible [1,15]. These mechanisms of resistance may be present in a single bacterial cell simultaneously, allowing for resistance to multiple antibiotic molecules [16]. Acquired resistance is of greatest concern, as it almost always results from the acquisition of ARGs from intrinsically resistant bacteria [13]. A prominent mechanism of propagating resistance from one bacterial cell to another is the acquisition of foreign ARGs through horizontal gene transfer (HGT), and these ARGs may then be cloned to subsequent daughter cells (vertical gene transfer). HGT accounts for the majority of AR that develops in previously susceptible bacteria [17].

### 1.2. Antibiotics and Antibiotic-Resistant Bacteria in Marine Environments

Marine ecosystems are home to the largest and most diverse communities of flora and fauna, presenting different habitats that cover 75% of the earth’s surface and harbour over 90% of all life on earth, including bacteria [18]. Marine-dwelling bacteria can be located in seawater (estimated at 10^6^ cells/mL), sediment on the seafloor (estimated at 10^8^ cells/mL), and on or within macroscopic organisms such as turtles, fish, and sea mammals [18,19,20]. Furthermore, the gastrointestinal systems of sea vertebrates represent a highly diverse microbial community. For example, a study on the gut bacterial flora in green sea turtles determined that there were over 174 identifiable bacterial families within the gastrointestinal system of these animals [12].

The dissemination of antibiotics and resistance-causing genes into marine environments is a major global concern, as it allows for the selection of antibiotic-resistant bacteria (ARB) within these highly concentrated and diverse bacterial populations [13]. Research efforts to address this problem have identified wastewater treatment plants (WWTPs) and run-off from agricultural industry as major contributors to the dissemination of antibiotics and resistant bacteria in marine environments [21,22]. Globally, influents into WWTPs and wastewater treatment processes vary greatly, and as a result, effluent of varying quality is discharged into highly diverse marine environments. From the AR point of view, most problematic sources of influents into WWTPs are hospital effluents, industry effluents, intensive agricultural practices, and domestic wastewater or sewage created by the general populace [22]. This type of waste is usually enriched with antibiotics and other antimicrobial agents, bacteria including antibiotic-resistant pathogens, and other biological molecules such as free plasmids and “naked DNA”, which may serve as vectors for ARGs and allow for HGT [23]. Collectively, WWTPs have a high propensity to generate, concentrate, and disseminate ARGs and MDRB in the immediate receiving marine environments [13,23]. It is worth noting, however, that different WWTPs across geographical locations employ different technologies to treat and process sewage; this may restrain the release of ARB and ARGs to varying degrees [24,25]. This issue is complicated further by the fact that policies that govern the implementation of wastewater treatment processes and technologies differ internationally or even regionally in some countries [23,26,27]. The global difference in WWTP policy and technology may allow some geographic areas to have a higher burden of AR pollution disseminated into their environment by WWTPs. Alternatively, countries with more restrictive policies and advanced treatment technologies may be capable of reducing AR from their influent sources through their wastewater treatment process.

Run-off from the agricultural industry that may not be directly associated with WWTPs is another important source of antibiotics, bacteria, and ARGs in marine environments. There is extensive antibiotic use in the agricultural industry of many countries not to just treat infections but also as growth promoters to pre-emptively prevent infections. This standard agricultural practice of long-term utilisation of broad-spectrum antibiotics can provide the perfect environment for cultivating ARB and propagating ARGs [21,28,29]. To put this into context, the cumulative global usage of antibiotics in agricultural facilities exceeds the usage in hospitals [28]. To counteract this problem, a few countries and particularly those in the European Union are now implementing stricter regulations on antibiotic use in agriculture. Nonetheless, this practice has persisted in many countries such as India, China, Spain, the USA, and Italy, that have a high agriculture turnover [30,31]. This exposure to different antibiotic compounds affects off-target bacterial populations, exerts intensive selection pressure within the microbiome of treated animals, and results in the shedding of ARB and antibiotics in manure, which then contaminates surface and groundwater [6,32]. This, too, creates AR “hot spots” providing pathways for contamination and indiscriminate spread of antibiotics, ARB, and ARGs in marine environments. It is therefore important to develop a robust, reliable, and universally applicable system for monitoring the spread of AR in marine ecosystems. Herein, we sought to evaluate sea turtles as sentinel species for monitoring as well as reporting incidences, dissemination, and distribution patterns of AR in marine environments.

## 2. Methods Used to Evaluate Turtles Are Sentinels for Monitoring AR in Marine Environments

### 2.1. Systematic Literature Search Strategy, INCLUSION and Exclusion CRITERIA, and Quality Assessment

**Figure 1:**
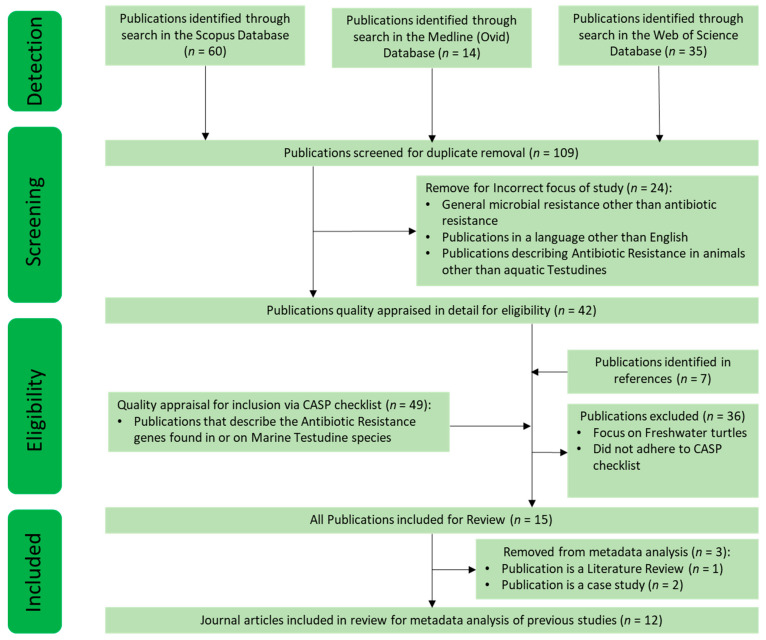
Summarised process for the literature search indicating detection, screening, and eligibility of included publications. Fifteen articles were retained for final analysis, but only 12 of these were direct, original studies on the abundance and effect of ARB in marine turtles. Twelve studies were included for analysis of the metadata.

Relevant literature was gathered systematically from three bibliographic databases: Medline (Ovid), Scopus, and Web of Science. The search terms used were: “Antibiotic Resistance Genes” or “Antibiotic Resistance”; and turtle or Testudines. This allowed for the identification of older documents analysing antibiotics by culture to obtain resistance identification, as well as newer documents identifying AR genes through metagenomic methods. Eligibility was not limited to publication type, including journal articles, reviews, and case studies. The eligibility criteria did not include language limitations, and coincidentally, all articles found to fit the eligibility criteria were publications in English. The exclusion criteria included papers that were not focused on the study of (or previous study of) AR in marine turtles. All used databases were searched from time of inception to December 2020. To allow for a critical review of the literature, subsequent citations from the results of the previously found articles were also appraised for suitability and inclusion in this review. Quality appraisal was ensured by using the CASP tool checklist relevant to each piece of literature, which allowed for the evaluation of the quality of each publication [33]. The literature search process is summarised in Figure 1.

### 2.2. Quantitative Evaluation of Compiled Data

All relevant data were extracted from text, figures, and tables. Data were then stratified according to predetermined variables including turtle species involved, anatomical sampling sites on turtles, the most prevalent bacterial isolates, classification of antibiotics against which AR was tested, geographical locations, and the potential source of AR. Quantitative data on AR were presented as mean percentages of observed AR in confirmed bacterial isolates. Where applicable, statistical comparisons between groups were done using the Kruskal–Wallis test, and *p* < 0.05 was considered significant.

## 3. Results and Discussion of Data on Using Turtles for Monitoring AR in Marine Environments

### 3.1. An Overview on the Systematic Search of the Literature on Using Sea Turtles for Monitoring AR

Fifteen published studies focusing on AR, ARB, and ARG isolated in sea turtle species were identified (Figure 1). Three out of fifteen studies were excluded from further analyses as two of these were case studies of specific antibiotic-resistant isolates in a singular diseased animal focusing on one antibiotic, and one was a review article. The remaining 12 publications were peer-reviewed journal articles describing studies on this theme; these were included in statistical analyses for the metadata. These were: [14,34,35,36,37,38,39,40,41,42,43,44].

### 3.2. Distribution of Studies on AR in Marine Environments by Geographical Site and Turtle Species

#### 3.2.1. AR in Turtles in Marine Environments and the Potential Influence of Geographical Location

**Figure 2:**
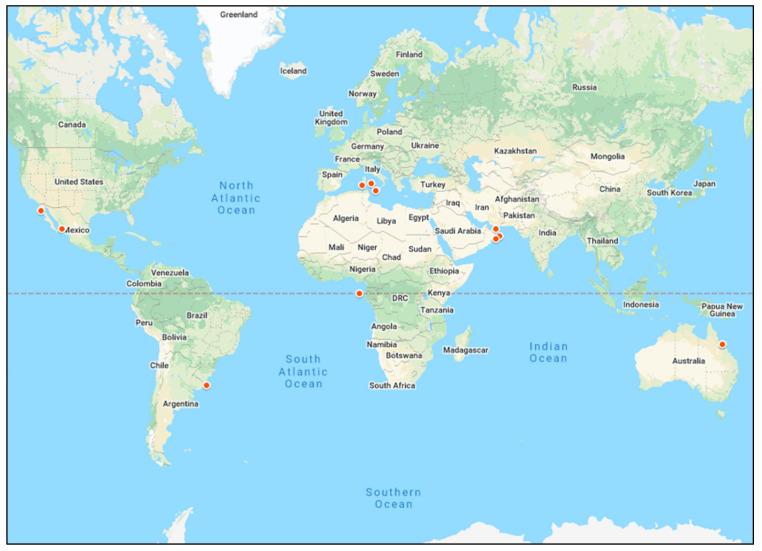
Map of geographical location and clustering of studies on the monitoring of AR in marine environments using sea turtles as an animal sentinel species (red pins). Represented areas are the Mediterranean and Arabian seas, the Pacific Ocean along the east coast of Australia and west coast of Mexico, and the South Atlantic Ocean along the west coast of Africa and the east coast of South America (modified from Google maps under the terms and conditions of the Creative Commons Attribution (CC BY) license).

The geographical distribution of marine turtles is worldwide with the exception of the Arctic and Antarctic waters [45]. Their highly migratory nature allows sea turtles to utilize foraging areas that often exceed thousands of kilometres during migration every few years [46]. To date, studies on the use of turtles to monitor AR in marine environments have examined at least five turtle species including green, loggerhead, leatherback, olive ridley, and hawksbill sea turtles, but the green and loggerhead turtles represent majority of these studies in vastly different geographical sites. While antibiotic usage rates and patterns of ARB also vary markedly across geographical areas [47], currently available studies on turtles only represent a very limited geographical distribution including the Mediterranean and Arabian seas, the Pacific Ocean along the east coast of Australia, and the South Atlantic Ocean along the west coast of Africa and the east coast of South America (Figure 2 and Table 1). Considering that wastewater through WWTPs and run-off from industry and agricultural waste are the major contributors to AR in marine environments, it is important to take regional policies of these AR “hot spots” into account when describing sources of pollution in different geographical areas.

The coastal areas of the Mediterranean and Arabian seas are shared by 21 cities and 6 nations representing Western Europe, the Middle East, and North Africa (Figure 2). These countries have marked variations in policies on antibiotic usage and efficiency in wastewater treatment, and up to 51% of the treated wastewater in countries around the Mediterranean and Arabian seas is recycled and utilised as agricultural irrigation [48]. On the Mediterranean coast, some European Union countries including Spain, France, Italy, Greece, Croatia, and Slovenia have implemented a One Health action plan against AR since 2017 [4]. These EU countries have also instituted high standards requiring the treatment of at least 71% of all wastewater through WWTPs before discharging into the sea [48]. However, the North African side of the Mediterranean Sea is much less stringent in regulating the general use of antibiotics and the discharge of wastewater through WWTPs [48]. This may further increase the propagation of AR by renewing the cycle of exposure to antibiotics, ARB, and ARGs [4]. This argument is further supported by the fact that similar AR patterns were observed in bacterial isolates from a WWTP in Oman and marine turtles in the Arabian Sea and the Gulf of Oman, suggesting that AR in marine turtles and wastewater effluent were directly linked [14,35,36]. Similar anthropogenic waste disseminated from WWTPs was described as the putative point source of ARB in turtles in the Mediterranean Sea in Italy [39,40] and the South Atlantic Ocean in Brazil [43]. This indicates that, for marine environments shared by markedly different jurisdictions and policies on environmental pollution, it is difficult to attain an accurate and reliable assessment of the geographical origins of AR. In these locations, monitoring of AR in sea turtles would simply indicate prevalence but not determine the point source or cause of AR.

On the east coast of the Pacific Ocean of Australia, AR was detected in sea turtle rehabilitation centres as well as wild sea turtles in surrounding areas such as the Cleveland Bay but no definitive and specific sources or causes of AR were identified [34,38]. It is notable however, that this site on the Pacific coast in Australia has many resident green turtles, inhabiting a receiving environment for effluents from WWTPs and it is adjacent to the Great Barrier Reef, one of the world’s heritage ecosystem. Given the extensive biological diversity represented by the Great Barrier Reef and its economic and cultural significance, local jurisdictions in this area (Townsville, Australia) have emphasised the need to determine specific sources and mechanisms by which AR has developed in this marine environment. To this effect, studies to screen for contamination with antibiotics and characterise the AR profile of the effluent from a WWTP that is adjacent to the Great Barrier Reef in Australia have been commissioned [49].

#### 3.2.2. AR in Marine Environments and the Potential Influence of Differences in Turtle Species

In all documented peer-reviewed studies on the use of turtles to monitor AR in marine environments herein, green turtles (*Chelonia mydas*) represented the majority (~75%) of the publications (Table 1). The complex life-history traits and dietary variations of green turtles make them particularly vulnerable to a wide range of anthropogenic threats, including AR pollution [11]. Dietary variations throughout a green turtle’s life, from omnivores as juveniles to herbivores as adults, along with their highly migratory nature exposes them to a diverse range of microbial communities and sources of pollution in different marine environments [46]. Based on the diversity in dietary requirements for sea turtles (summarised in Table 1), different sea turtle species may represent the level of contamination throughout their food chains thus providing information on the prevalence and relative abundance of ARB and ARGs in their food sources. It is conceivable that marine turtle species, like the leatherback (*Dermochelys coriacea*), which feeds almost exclusively on jellyfish, may accumulate antibiotics, ARGs, and ARB from any area in which the food source has travelled. Thus, turtle species depending on a more protein-rich diet may not represent AR profiles within their immediate environment, but possibly other geographical regions. In contrast, more mature green sea turtles primarily feeding on seagrasses and algae in times of non-migration are likely to provide more accurate and site-specific information on the bioaccumulation of antibiotics, ARGs, and ARB due to the stationary nature of seagrass and algae within near-shore environments. Certainly, the ability of seagrass to bioaccumulate other chemical compounds and pollutants has been documented previously [50]. However, distinguishing the time of last migration in adult green turtles is not possible without constant and costly satellite tracking, and this was not noted in any of the literature studied for this review. Furthermore, some studies specifically utilised migrating and breeding adult turtles and specifically egg-laying females for research purposes [14,36,37]. Though this class of adults is easier to capture when in high numbers at breeding and egg-laying sites, the nature of migration patterns for this age group indicates that detected AR within their microbiome may not represent the AR profile for the site of location. It is therefore more likely that the AR profile within adult turtles of a migratory age (i.e., sexual maturity) represents any location between their residential feeding grounds and their breeding sites; this may be up to 2600 kilometres long, containing any number of possible AR pollution dissemination points [35]. In contrast, however, a sub-adult class of green turtles may offer a more effective method for assessing AR within a specific geographical area. The juvenile class of green turtles are usually resident to a particular area, have the same diet predominantly comprised of seagrass as adults, but have not reached sexual maturity and therefore do not migrate [51,52]. However, in the seven studies identified for this review, age class and migration status were not consistently defined for all the green sea turtles sampled [14,34,35,36,38,43,44]. In two of these identified studies [34,43], juvenile as well as adult green sea turtles were sampled but the AR profiles were not stratified and reported according to the age class of the animals. The potential for a correlation between the microbiome and AR profiles with age and sexual maturity may also be true for other marine turtle species. However, due to declining populations and the lack of adequate ecological data, much less is known about complex life-history traits of many different turtle species.

Bacteria can colonise different anatomical regions of the turtle including the respiratory and reproductive tracts, the plastron or carapace, and more predominantly, the gastrointestinal tract [53]. It is also likely that the dietary requirements for different turtle species determine the microbiota composition in their gastrointestinal systems. This factor is pertinent in monitoring AR using different turtle species because anatomic sites involved in the gastrointestinal tract are often utilised, such as cloacal or oral cavity swabs (Table 1). It was argued that ocular surface samples of turtles more accurately represent the surrounding environment, not affected by feeding or migratory habits [38]. Similarly, analyses of freshly hatched eggs and oviduct fluid may reflect the ability of marine turtles to serve as vectors for vertical transmission of AR from mother to hatchling and consequently serving as a carrier and spreader of AR in marine environments worldwide [14,37]. These observations indicate that it is rational to recommend that any analyses on the effects of exposure to antibiotics and ARB should account for resident bacteria in anatomical sampling sites, turtle species in question, and geographical locations to ascertain significance.

### 3.3. Detection and Quantification of AR Profiles in Marine Environments

#### 3.3.1. Present Challenges in Detecting ARB in Marine Turtles

The identification of ARB in biological samples obtained from turtles in marine environments is heavily dependent on our knowledge of the detectable resident microbiome in the different turtle species and more importantly, the methods used to isolate, detect, and characterise ARB. In all published studies on AR in sea turtles to date, resistance has been identified in at least 48 different bacterial species. Gram-negative coliforms and particularly *Citrobacter* spp were the predominant bacteria representing 25% of all identified isolates. *Citrobacter* spp constitutes part of the normal gut flora of sea turtles but it has also been associated with severe infections in humans as well as captive and wild sea turtles [12,39,41,54]. Sea turtles were also found to harbour other ARB such as *Salmonella* spp and *Pseudomonas* spp, representing an average of 11% and 16% of isolated ARB [14,35,37,39] (Table 1). *Salmonella* spp and *Pseudomonas* spp have the potential to cause severe zoonotic infections that are also resistant to treatment in humans. Indeed, severe salmonellosis associated with the consumption of turtle meat or eggs in some coastal locations has been documented previously [40,55]. Zoonotic transmission of *Pseudomonas* spp. from turtles to humans linked to diseases including urinary and respiratory infections, meningitis, and endocarditis has also been reported [56]. From a One Health perspective, the proliferation of these types of ARBs within marine turtles can have equally detrimental effects on the growth, maturation, and overall health of the host [18,57,58]. Additionally, pathogenic ARB in sea turtles might signify a risk for other marine creatures and especially through predation [37,42]. This provides a mechanism for spreading ARB throughout the food chain [59,60,61]. All these studies indicate that sea turtles are a good sentinel model for monitoring AR including the detection of potentially pathogenic ARB in marine environments. However, the true magnitude of ARB in entire microbiomes in turtles (resistome) remains unknown.

In all documented studies to date (Table 1), identified common and predominant bacterial isolates were used to detect AR, but these are not representative of entire resistomes likely to be hosted and disseminated by sea turtles. This is primarily because all studies in this area have utilized many different methods to isolate and characterize ARB. Culturing bacteria followed by antibiotic sensitivity testing was the most used method for monitoring AR in sea turtles. This approach limits the discovery of ARB to only bacteria that grow under laboratory conditions, thus creating a bias that may over-represent specific genera of bacteria such as *Citrobacter*, *Pseudomonas*, *Enterococcus*, and *Vibrio*. It has been shown that microbiomes identified via culture methods account for only ~1% of the true microbial diversity [62]. This is especially true for animals inhabiting marine environments where diverse bacterial communities exist [63]. This problem is likely to be resolved by extensive application of modern molecular biology methods such as sequencing 16 s ribosomal RNA gene and metagenomic sequencing of entire microbiomes in identifying ARB and ARGs in marine environments [64]. However, these newer approaches require relatively expensive facilities and highly skilled technical expertise but may start to become more readily available with advancements in these technologies. For this review, because of the outlined methodological limitations associated with isolating bacteria and identifying AR in sea turtles, reported frequencies and the distribution patterns of ARB were not stratified and analysed by genus or species of the bacterial isolates.

#### 3.3.2. Quantitative Evaluation of Phenotypic AR Patterns in Marine Turtles

**Figure 3:**
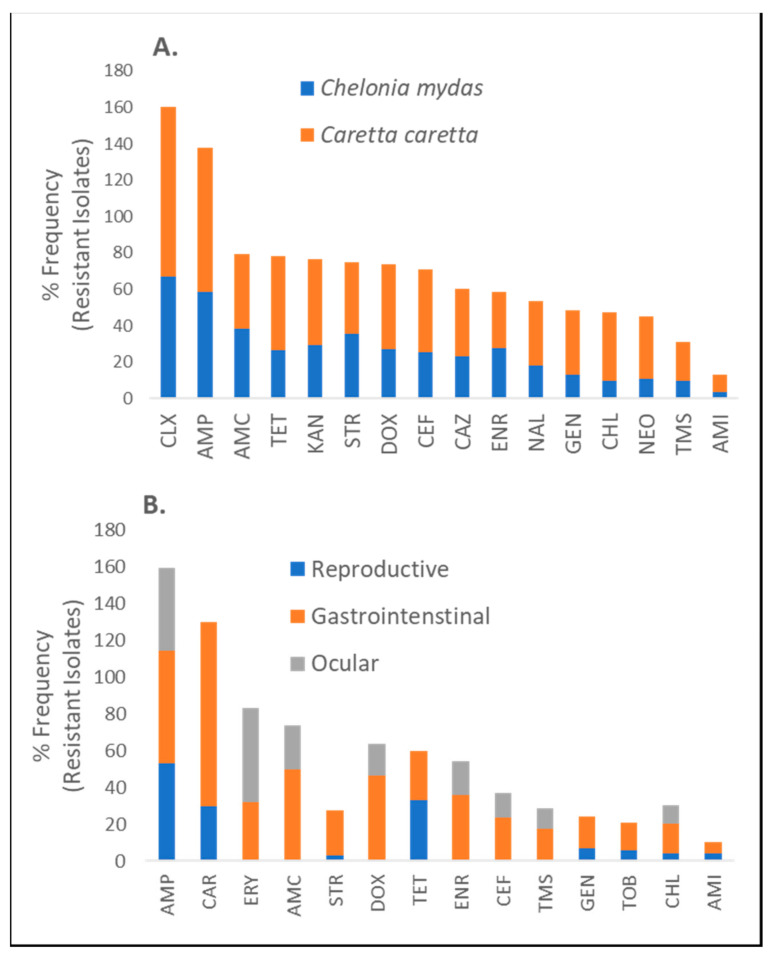
Frequency distribution of AR against commonly used antibiotic drugs stratified by turtle species (*Chelonia mydas* and *Caretta caretta*) irrespective of geographical marine environment, biological specimen considered, or tested bacterial isolates (**A**) and frequency distribution of AR stratified by considered biological specimen irrespective of geographical marine environment, turtle species, or tested bacterial isolates (**B**). Abbreviations are defined as: AMI = Amikacin; AMO = amoxicillin; AMC = Amoxicillin-clavulanic acid; AMP = Ampicillin; CAR = Carbenicillin; CAZ = Ceftazidime; CEF = Ceftiofur; CLX = Cephalexin; CHL = Chloramphenicol; DOX = Doxycycline; ENR = Enrofloxacin; ERY = Erythromycin; GEN = Gentamicin; KAN = Kanamycin; NAL = Nalidixic acid; NEO = Neomycin; STR = Streptomycin; TET = Tetracycline; TOB = Tobramycin; TMS = Trimethoprim-sulfamethoxazole.

**Figure 4:**
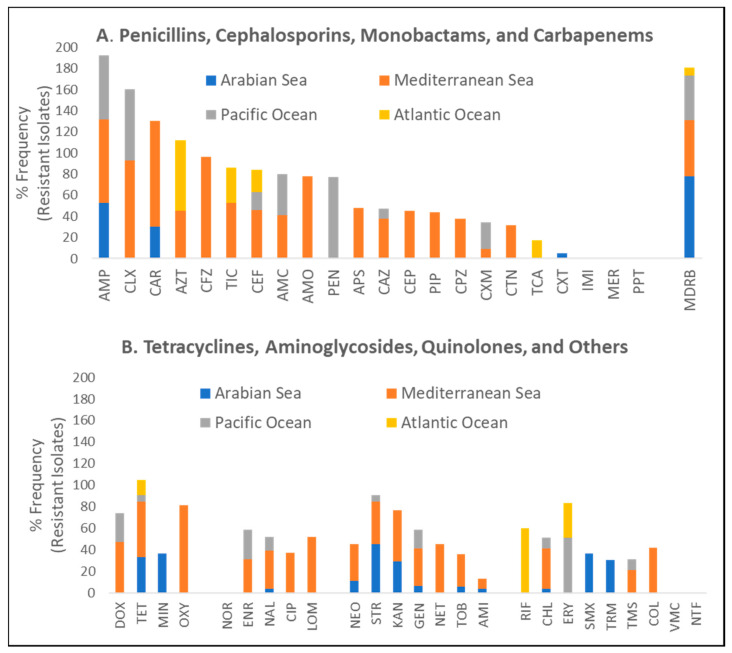
Frequency distribution of AR against commonly used antibiotics stratified by geographical marine environment irrespective of biological specimen considered, bacterial isolates, or turtle species for beta-lactams (**A**) and other antibiotic classes (**B**). Abbreviations are defined as: AMI = Amikacin; AMO = Amoxicillin; AMC = Amoxicillin-clavulanic acid; AMP = Ampicillin; APS = Ampicillin-salbactam; AZT = Aztreonam; CAR = Carbenicillin; CAZ = Ceftazidime; CEF = Ceftiofur; CEP = Cefepime; CFZ = Cefazolin; CIP = Ciprofloxacin; CLX = Cefalexin; CHL = Chloramphenicol; COL = Colistin; CTN = Ceftriaxone; CXM = Cefotaxime; CXT = Cefoxetin; DOX = Doxycycline; ENR = Enrofloxacin; ERY = Erythromycin; RIF = Rifampicin; GEN = Gentamicin; IMI = Imipenem; LOM = Lomefloxacin; MDRB = percentage of multi-drug resistant isolates; MER = Meropenem; MIN = Minocycline; KAN = Kanamycin; NAL = Nalidixic acid; NET = Netlimicin; NFT = Nitrofurantoin; NEO = Neomycin; NOR = Norfloxacin; PEN = Penicillin; OXY = Oxytetracycline; PIP = Piperacillin; PPT = Piperacillin-tazobactam; STR = Streptomycin; SMX = Sulfamethoxazole; TCA = Ticarcillin-clavulanic acid; TET = Tetracycline; TIC = Ticarcillin; TOB = Tobramycin; TRM = Trimethoprim; TMS = Trimethoprim/sulfamethoxazole; VMC = Vancomycin.

To identify and quantify the functional nature of acquired AR in sea turtles in marine environments, our analyses revealed that tests have been done against all major antibiotic classes including beta-lactams, tetracyclines, aminoglycosides, and quinolones. Only two turtle species (green and loggerhead) were represented by multiple studies at different geographical sites (Table 1). Irrespective of geographical site, biological specimen, and isolated bacteria, there were no statistically significant differences in reported frequencies of resistance against all tested antibiotics stratified by turtle species (Figure 3A). Similarly, no differences in reported frequencies of resistance were observed when AR profiles were stratified by the anatomical origin of the specimen for bacterial isolation (Figure 3B). It is important to note, however, that these statistical analyses may not have the required power to detect differences because of the limited number of studies as well as a low number of tested turtles. Our analysis of the global distribution frequencies shows that AR in turtles in marine environments is overrepresented by beta-lactams (penicillins, cephalosporins, monobactams, and carbapenems) by a two-fold difference compared to all other antibiotic classes. However, resistance was reported against all major antibiotic agents (Figure 4). Furthermore, it should be noted that the majority of specimen data utilised within this review were obtained from the Mediterranean and Arabian Seas. This may provide a biased point of view towards these geographical areas thus creating a need for more studies in unrepresented locations.

**Figure 5:**
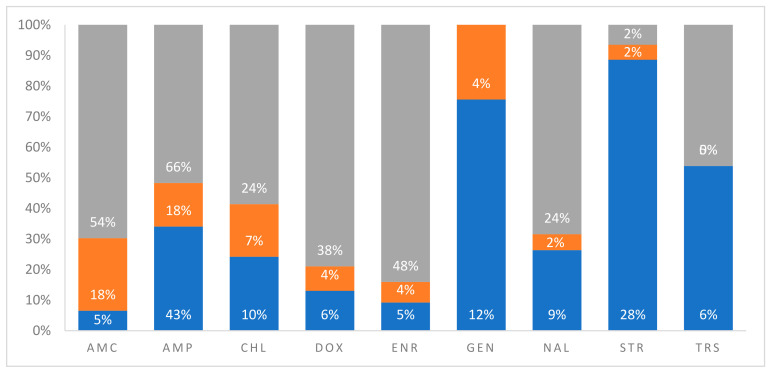
Percentage distribution frequency for AR to common antibiotics in bacteria isolated from sea turtles in different marine environments and stratification by potential sources of exposure as postulated by each original study. This includes rehabilitation centres for sick or injured turtles (grey), treated wastewater from WWTPs (blue), and other unidentified sources of anthropogenic pollutants (orange). Abbreviations are defined as: AMC = amoxicillin-clavulanic acid; AMP = ampicillin; CHL = chloramphenicol; DOX = doxycycline; ENR = enrofloxacin; GEN = gentamicin; NAL = nalidixic acid; STR = streptomycin; TRS = trimethoprim-sulfamethoxazole.

While not all antibiotics were tested for AR in all reported studies included in this review, there was no apparent difference in reported frequencies of resistance to commonly used, first-line drugs such as ampicillin, which was tested at multiple sites. Moreover, a relatively similar incidence of MDRB across all major antibiotic classes was observed in all represented geographical locations in the currently available studies (Figure 4). The analysis of these data, when stratified by geographical area, further suggests that the predominant age class of marine turtle that has been utilised for these previous studies (i.e., adults that are capable of migration, or currently migrating) may not adequately represent AR within an immediate area unless assurances can be made towards its residency and time of last migration. Furthermore, the occurrence of resistance to reserved antibiotic classes such as quinolones and aminoglycosides is truly worrying as these drugs are usually the last line of defence in many life-threatening infections. Collectively, these data seem to suggest that, irrespective of geographical location, selection pressures that have driven the development of AR in sea turtles and marine environments are relatively similar. Future studies in this area should focus on identifying AR and ascertaining definitive sources for AR. It is also of significant note that the current literature in this field has not directly correlated prevalence in AR in sea turtles and marine environments with direct indices of contamination with specific antibiotics. Addressing these gaps in knowledge will be instrumental in devising mitigation strategies. In the current study, we were able to stratify the frequencies of observed AR with putative sources of antibiotics, ARGs, and ARB as predominantly defined by the primary authors of included studies. We show that the highest prevalence of MDRB may be associated with sources such as WWTPs and rehabilitation centres for sick and injured sea turtles, which are both considered to be sources of exposure to antibiotics and ARB (Figure 5).

#### 3.3.3. Quantitative Evaluation of Anthropogenic Sources of AR in Marine Turtles

On average, WWTPs and turtle rehabilitation centres accounted for 58% and 49% of MDRB isolates from sea turtles in all published studies. This result is not surprising because the rehabilitation of sick or injured turtles for purposes of conservation is usually associated with marked broad-spectrum antibiotic usage, as methods for rapid diagnosis for wild animals are sorely lacking. Antibiotics classified as quinolones (nalidixic acid and enrofloxacin), beta-lactams (ampicillin and amoxicillin-clavulanic acid), and tetracyclines (doxycycline) showed the highest frequency of resistance that potentially originated from WWTPs and turtle rehabilitation centres. This directly correlates with the increased usage of these antibiotic classes at rehabilitation centres because they have a broad spectrum of activity and are safe to use in reptiles. These arguments on the observed AR patterns are supported by the fact that there was no resistance against aminoglycosides (gentamicin and streptomycin) when tested for at turtle hospitals or rehabilitation centres. Coincidentally, aminoglycosides are severely nephrotoxic in reptiles, and as such, the application of this class to treat infections in turtles is very limited. In contrast, chloramphenicol is not a drug of primary choice in treating sick turtles, but it showed a relatively high frequency of resistance. This may be attributable to generalized non-specific mechanisms of resistance such as the increased expression of efflux pumps by bacteria.

The AR pattern associated with WWTPs or other unidentified sources was more diverse, affecting all the major antibiotic classes. It is plausible that this is because WWTPs are points of convergence for a diverse mix of antibiotics, ARGs, MDRB, and other pollutants that may select for AR. However, specific sources and mechanisms contributing to observed AR patterns in turtles in marine environments are not known. It is reasonable to assume, however, that similar pools of ARGs and means of AR acquisition seen in other ecosystems are involved here as well. For instance, the respective genes encoding for some of the reported resistance patterns against tetracyclines (*tetA*), sulphonamides (*sulII)*, beta-lactams (*blaTEM* and *blaCTXM*), and quinolones (*qnrS*), were also isolated in wild sea turtles [37]. In addition, a higher percentage of the *int1* gene, encoding for the mobile element class 1 integron has also been identified in bacteria isolates from sea turtles [37]. This suggests a propensity for HGT of ARGs to enhance the spread of ARB in sea turtles in marine environments.

## 4. Conclusions

This review evaluates the use of sea turtles as a sentinel species for monitoring as well as reporting incidences, dissemination, and distribution patterns of AR in marine environments. The extensive geographical distribution and relatively long lifespan exhibited by sea turtles allow for their utilisation as a sentinel species for AR surveillance in marine environments. However, the majority of studies originated from the Mediterranean and Arabian Seas, suggesting a bias in previous research towards these geographical areas, likely due to the accessibility of turtles in these regions during mating and migration. To date, five marine turtle species including green, loggerhead, leatherback, olive ridley, and hawksbill have been used in studies on monitoring AR in marine environments. While there were no statistically significant differences in reported frequencies of AR when data were stratified by turtle species or anatomical origin of the tested specimen, it can be hypothesised that turtle species with higher protein-rich diets may contain AR elements that are representative of their mobile food sources. Conversely, species that rely on more stationary food sources may provide a more accurate representation of their immediate feeding grounds. In monitoring the prevalence of AR in marine environments using sea turtles, the majority of studies utilised adult animals capable of, or during active, migration. This means that identified microbiomes and associated AR profiles may only indicate the existence of AR in respective marine environments, without a clear indication of specific geographical sources of the problem. The sub-adult class of green sea turtles may be a more effective animal model for assessing AR in specific geographical locations and thus recommend that future AR surveillance studies should use the non-migratory juvenile stage of green sea turtles.

Furthermore, the true magnitude of bioavailable antibiotics and ARB in entire microbiomes in marine turtles has remained unknown. Herein, we show that the bulk of knowledge on surveillance of AR in marine environments using turtles has relied on culture and antibiotic sensitivity tests with the identification of phenotypic AR traits. These data are not likely to represent the entire resistomes for all ARB in tested microbiomes due to the existence of bacteria that is not culturable under ordinary laboratory conditions. In addition, our knowledge of the prevalence of specific ARGs and other mechanistic determinants of the phenotypic AR exhibited by sea turtles in marine environments is very limited. There is therefore a need to determine if ARGs and ARB profiles in sea turtles in marine environments are associated with similar profiles in specific anthropogenic AR “hot spots” such as WWTPs. Despite all these gaps in knowledge, analyses in this review show that there is a predominance in resistance to the beta-lactam class of antibiotics and this reflects the extensive usage of this class of antibiotics globally. Nonetheless, MDRB and resistance against less commonly used antibiotics including quinolones and aminoglycosides was also detected in sea turtles. This presents a serious threat to environmental and public health. Future studies should concentrate on the accurate determination of point sources, specific causes, and mechanisms, as well as prevalence and distribution patterns of AR in marine environments. The acquisition of this knowledge will be critical in designing effective mitigation strategies.

## Figures and Tables

**Table 1:** Global distribution of geographical sites where different sea turtle species have been examined as sentinel species for AR. The plus sign (+) denotes the isolation of multi-drug-resistant bacterial isolates.

Turtle Species	Food Sources in Marine Environments	Geographical Site & Presumed ARB Source, Biological Sample, and Dominant Bacterial Isolate	MDRB
Green (*Chelonia mydas*)	Juveniles: Shrimp, crabs, clams, mussels, fish, squid.Adults: Seagrass, algae	NW Indian Ocean & Arabian sea, fresh eggs, *Psuedomonas* spp, (*n* = 30). [14]	+
		Gulf of Oman Arabian sea, oviductal fluid, *Citrobacter* spp, (*n* = 40). [35]	+
		Gulf of Oman Arabian sea, oviductal fluid, *Citrobacter* spp, (*n* = 20). [36]	+
		Pacific Ocean (Rehabilitation centres, NE Australia), ocular swabs, *Vibrio* spp, (*n* = 7). [38]	+
		Atlantic Ocean (South coast of Brazil), cloacal or rectal swabs, *Enteroccus* spp, (*n* = 6). [43]	+
		Pacific Ocean (Baja California & Sinaloa Mexico), nasopharyngeal & oral swabs, *Vibrio* spp, (*n* = 42). [44]	+
		Pacific Ocean (Rehabilitation centres & wild, NE Australia), cloacal swabs, *Citrobacter* spp, (*n* = 73). [34]	+
Loggerhead (*Caretta caretta*)	Juveniles & adults: Shrimp, crabs, clams, mussels, fish, squid.	Mediterranean Sea (Rehabilitation centre in Italy), internal organs, cloacal, oral & skin swabs, *Aeromonas* & *Citrobacter* spp, (*n* = 20). [37]	+
		Central Mediterranean Sea (Italy), cloacal, oral & skin swabs, *Proteus* & *Citrobacter* spp, (*n* = 19). [39]	+
		West Mediterranean Sea (Italy), oral & cloacal swabs, *Pseudomonas* & *Citrobacter* spp, (*n* = 35). [42]	+
Leatherback (*Dermochelys coriacea*)	Juveniles & adults: Jellyfish, other soft-bodied animals, algae.	Atlantic Ocean (Gulf of Guinea, Principe Island), oral & cloacal swabs, *Pseudomonas* spp, (*n* = 10 Leatherback & *n* = 2 Green). [40]	-
Olive Ridley (*Lepidochelys olivacea*)	Juveniles & adults: Shrimp, crabs, clams, mussels, fish, squid.	Pacific Ocean (Sinaloa Mexico), nasopharyngeal & oral swabs, *Vibrio* spp, (*n* = 22). [44]	+
Hawksbill (*Eretmochelys imbricate*)	Juveniles and adults: Sponges, squid, shrimp, anemones.	Atlantic Ocean (South coast of Brazil), cloacal or rectal swabs, *Enteroccus* spp, (*n* = 2). [43]	+

## Data Availability

All data associated with this work are provided herein.
